# Significant Activity of Pytren-2Q, a 2‑Quinoline Polyamine Compound, against High-Concern Human Pathogenic Fungi

**DOI:** 10.1021/acsomega.5c09700

**Published:** 2026-02-06

**Authors:** Mario Inclán, Maria Paz Clares, Eduardo Álvarez-Duarte, Fabiola Fernández-Silva, Valentina Salas, Josep Guarro, Javier U. Chicote, Begoña Verdejo, Estefanía Delgado-Pinar, Enrique García-España, Antonio García-España, Enrique Calvo

**Affiliations:** † Molecular Science Institute, Universitat de València, C/Catedrático José Beltrán 2, 46980 Paterna, Spain; ‡ Departamento de Farmacia, Facultad de Ciencias de la Salud, Universidad CEU Cardenal Herrera, Carrer Santiago Ramón y Cajal, 20, 46113 Alfara del Patriarca, Spain; § Laboratorio Micología, ICBM−F. de Medicina, 14655 Universidad de Chile, 1025000 Santiago, Santiago Metropolitan Region, Chile; ∥ Instituto Microbiología Clínica, Facultad de Medicina, 28040 Universidad Austral de Chile, Independencia 631, 5110566 Valdivia, Chile; ⊥ Mycology Laboratory, Biomedical Department, Public Health Institute of Chile, Av. Marathon 1000, 7780050 Santiago, Chile; # Unitat de Microbiologia, Departament de Ciències Mèdiques Bàsiques, Facultat de Medicina i Ciències de la Salut, 16777 Universitat Rovira i Virgili and Institut d’Investigació Sanitària Pere Virgili (IISPV), Carrer Sant Llorenç, 21, Reus, 43201 Tarragona, Spain; ∇ Departament de Medicina i Cirurgia, Institut d’Investigació Sanitària Pere Virgili (IISPV), Servei Anatomia Patològica, Hospital Universitari Joan XXIII Universitat Rovira i Virgili (URV), Mallafré Guasch, 4, 43007 Tarragona, Spain; ○ Departamento de Bioquímica y Biología Molecular, Universitat de Valencia, Carrer del Dr. Moliner, 50, 46100 Burjassot, Spain; ◆ Department of Biochemistry and Biotechnology; Institute of Health Research Pere Virgili (IISPV); Center of Environmental, Food and Toxicological Technology (TecnATox), Universitat Rovira i Virgili, C/Marcel·lí Domingo 1, 43007 Tarragona, Spain

## Abstract

In view of the worldwide expansion of life-threatening invasive fungal diseases (IFDs) and fungal drug resistance, new antifungal drugs are urgently needed. To guide research and public health policies, the World Health Organization (WHO) specified 19 priority-concern human mold and yeast pathogens associated with serious risk of mortality or morbidity. We assessed the *in vitro* susceptibility of twenty-three fungal pathogens, 13 of them included in the WHO priority concern list, to a set of 24 polyamine derivatives generated by linking either alkylated ethylenediamine or a polyamine macrocycle to heterocycles, such as pyridine or quinoline, or polycyclic aromatic compounds, such as anthracene, pyrene, or fluorene. Here we report strong *in vitro* antifungal activity of a compound generated by linking 2-quinoline to a pyridinophane macrocycle (Pytren-2Q). Pytren-2Q was particularly active against pathogenic molds including WHO critical priority wild-type and azole-resistant *Aspergillus fumigatus*. These results, in addition to low toxicity, water solubility, and ease of production of Pytren-2Q, suggest that this compound could be of therapeutic interest and may be worth future investigation and validation.

## Graphical abstract

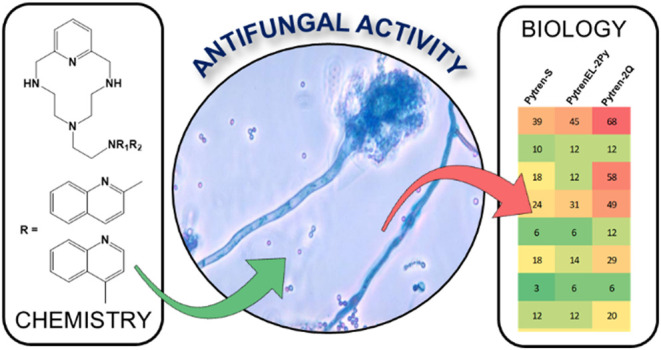

## Introduction

Fungal infections could range from common superficial infections, such as athlete’s foot or nail infections, to life-threatening invasive diseases caused by yeast like *Candida albicans* or molds such as *Aspergillus fumigatus*.^1,2^ Both yeasts and molds are microscopic fungi but, while yeasts are single-celled and reproduce by budding, molds form multicellular filaments known as hyphae that grow by apical extension.^3^ In the last decades, invasive fungal diseases (IFDs) have been steadily increasing worldwide causing over 1.5 million early deaths a year, a ratio similar to that of tuberculosis and three times higher than that of malaria.^4,5^ Concerned about this scenario and to guide research and public health policies, the World Health Organization (WHO) released in 2022 its first fungal priority pathogens list, which specifies the 19 most common human yeasts and mold pathogens associated with serious risk of mortality or morbidity.^6,7^ The most dangerous group (Critical group) consists of just four pathogens *C. albicans*, *A. fumigatus*, *Cryptococcus neoformans*, and *Candida auris*.^6–8^

Fungal pathogens, around 200 species of yeasts and molds out of more than 150,000 described fungi species, do not normally cause serious harm in immunocompetent individuals and even form part of our healthy gut microbiota, like the yeast *C. albicans*. However, due to their opportunistic nature, they may become invasive in individuals with a weakened immune system such as in the increasing number of cancer patients being treated with chemotherapy and radiation therapy, organ transplant recipients, and individuals suffering from immune debilitating diseases such as AIDs.^9,10^ Moreover, IFDs expansion has been facilitated by the emergence of resistance to current fungal treatments driven by the massive and indiscriminate use of antifungal products in agriculture, since the traits and genetic elements of fungi virulence, as suggested by the opportunistic nature of fungi, evolved independently of animal infections.^11^

On the other hand, development of antifungal medicines is a difficult task due to the scarcity of fungal drug targets as fungi and human biomolecules are highly similar. Despite their enormous differences in phenotype, ecology, and natural history, fungi are evolutionarily the closest related group to animals (*metazoa*).^12^ In this sense, only a small number of antifungal drug types exist in the clinic (azoles, echinocandins, pyrimidines, and polyenes) and only a few others are currently under development.^13–15^ Thus, the development of novel antifungal compounds is still highly needed.

In recent years, we have reported that some synthetic polyamine derivatives and/or their metallic complexes have activity as antioxidants or antiparasitic in various biological settings such as murine models of systemic inflammation (bacterial-induced endotoxemia) or parasitic (acute-phase Chagas disease).^16–20^ These compounds belong to two families of synthetic polyamines generated by linking heterocycles, such as pyridine or quinoline, or polycyclic aromatic compounds, such as anthracene, pyrene, or fluorene, to either alkylated ethylenediamine (L1–6) or a polyazamacrocycle known as Pytren (L7–24) (Figure 1). The name Pytren derives from the fact that it is obtained from the cyclization of 2,6-bis-bromomethyl-pyridine with tris­(2-aminoethyl)­amine (tren*)*.

**Figure 1:**
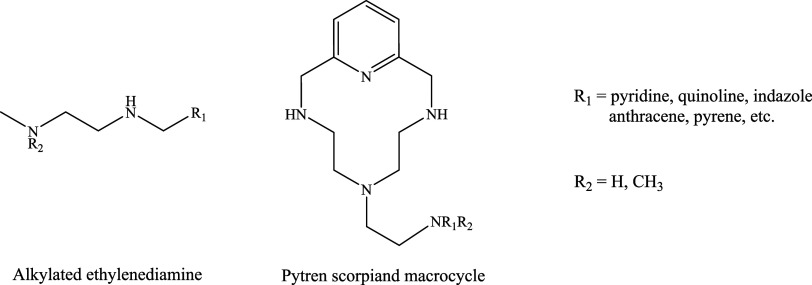
Schematic representation of the two families of compounds studied here.

Thus, since polyamines and nitrogen-containing heterocycles like pyridine and quinoline are often moieties of antifungal compounds,^21–24^ we decided to test if some of the above synthetic polyamine derivatives could have antifungal activity. To do so, we checked 22 reported^16,17,19,20^ and 2 newly synthesized and recently submitted polyamine derivatives using clinical isolates of yeasts and molds belonging to 23 different fungal pathogens, 13 of which are included in the WHO 19 fungal pathogens priority list.^6^

In this study, we provide evidence of *in vitro* antifungal activity of Pytren derivatives, especially Pytren-2Q, a Pytren scorpiand macrocycle linked to a 2-quinoline moiety, which showed potent antifungal activity particularly against pathogenic molds including WHO critical priority, wild-type (WT), and azole-resistant, *A. fumigatus*.

## Results and Discussion

### Antifungal Activity Screening

All compounds were preliminarily screened for antifungal activity by a disc diffusion procedure. Antifungal activity was determined against 8 clinical isolates (6 yeasts and 2 molds) as the diameter of the growth inhibition zone measured in mm (Figure 2). The test showed that while almost all Pytren derivatives (compounds L7–24), with the only exception of compound L24, have antifungal activity, alkylated ethylenediamine derivatives (compounds L1–6) had no activity. A Pytren macrocycle without the aliphatic arm (Pyclen–OH) used as the control was also inactive. The most active compounds, L12 (Pytren-2Q) followed by L10 (PytrenEl-2Py), L21 (Pytren-S), L7 (Pytren), and L15 (Pytren-2QI), were selected for extended antifungal evaluation against twenty-three pathogenic yeasts and molds.

**Figure 2:**
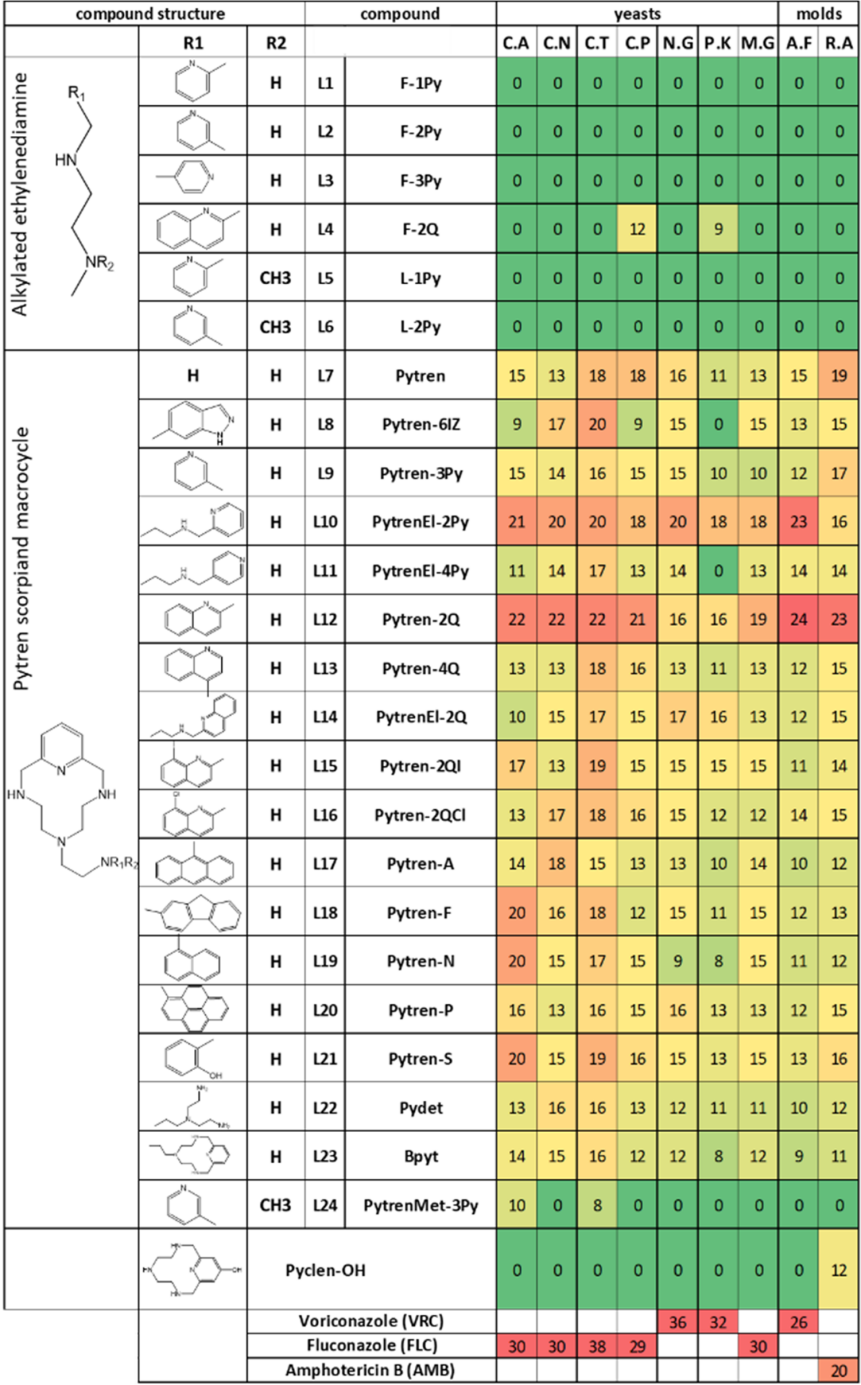
Fungal growth inhibition and molecular structure of polyamines L1–L24. Heat map representing color-coded fungal growth zone inhibition (zone diameter numerical values in millimeters inside cells) from strong green (no inhibition) to strong red (maximum inhibition) within each fungal pathogen. Yeasts: *C. albicans* (C.A), *C. neoformans* (C.N), *Candida tropicalis* (C.T), *Candida parapsilosis* (C.P), *Nakaseomyces glabratus* (formerly *Candida glabrata*) (N.G), *Pichia kudriavzevii* (formerly Candida krusei) (P.K), and *Meyerozyma guilliermondii* (formerly *Candida guilliermondii*) (M.G). Molds: *A. fumigatus* (A.F), and *Rhizopus arrhizus* (formerly *Rhizopus oryzae*) (R.A). Voriconazole, Amphotericin B, and Fluconazole were used as control antifungal drugs.

### Activity of Pytren-2Q against Pathogenic Yeasts

We further tested the activity of Pytren, Pytren-S, PytrenEl-2Py, Pytren-2QI, and Pytren-2Q against 28 clinical isolates of yeasts by using a specific yeast microdilution procedure to calculate their minimum inhibitory concentrations (MICs) (Figure 3 and ESI Table S1). Those 28 clinical isolates, including a *N. glabratus* strain resistant to azoles, belong to 11 different yeast fungal pathogens, 7 of them included in the WHO list of priority fungal pathogens. In addition, an American Type Culture Collection (ATCC) strain of *C. parapsilosis* was used as a control. The assay showed that, although all compounds were able to completely inhibit yeast growth, their MIC values were higher than those of the control drug voriconazole in each case, except for azole-resistant *N. glabratus*, which was around eight times more sensitive to the compounds (MIC values between 1 and 2 μg/mL) than to voriconazole (MIC = 8 μ/mL). Like in the disc diffusion assay, the most effective compounds were Pytren-2Q, followed by PytrenEl-2Py and Pytren-S. The most active compound, Pytren-2Q, showed the closest range of activity to voriconazole, between 19 and 68% of voriconazole antifungal activity for the WHO priority group of yeasts included in the assay.

**Figure 3:**
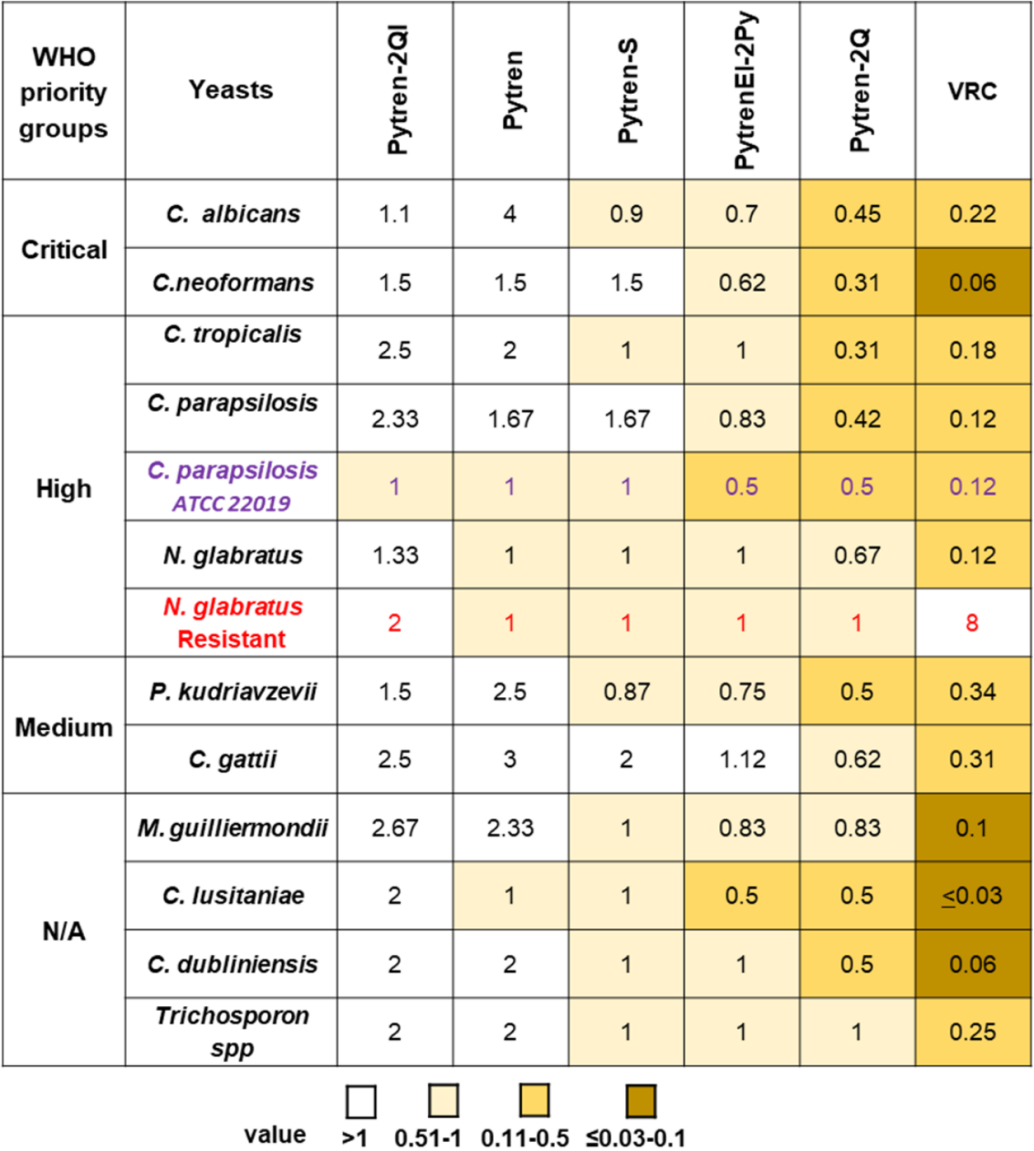
Antifungal activities of selected Pytren macrocyclic polyamines against human pathogenic yeasts. Numbers inside the boxes represent the minimum inhibitory concentrations (MICs) in μg/mL of selected compounds Pytren, Pytren-S, PytrenEL-2Py, Pytren-2QI, and Pytren-2Q against pathogenic yeasts. Yeast strains are sorted into critical, high, and moderate priority groups, as in the WHO list of fungal pathogens. A heat map was generated with colors ranging from MIC values >1 (white) to ≤ 0.03–0.1 (dark brown). Voriconazole (VRC) was used as a control antifungal drug.

Curiously, sensitivity and resistance to Pytren-S, PytrenEl-2Py, and Pytren-2Q were strain specific, with *P. kudriavzevii*, *C. tropicalis*, and *C. albicans* being the more sensitive fungal pathogens. *Clavispora lusitaniae* (formerly *Candida lusitaniae*), *Candida dubliniensis*, and *M. guilliermondii* were the more resistant. Interestingly, phylogenetically close fungal pathogens like *C. albicans* and *C. dubliniensis* showed very different sensitivity to these compounds (Figure 4).

**Figure 4:**
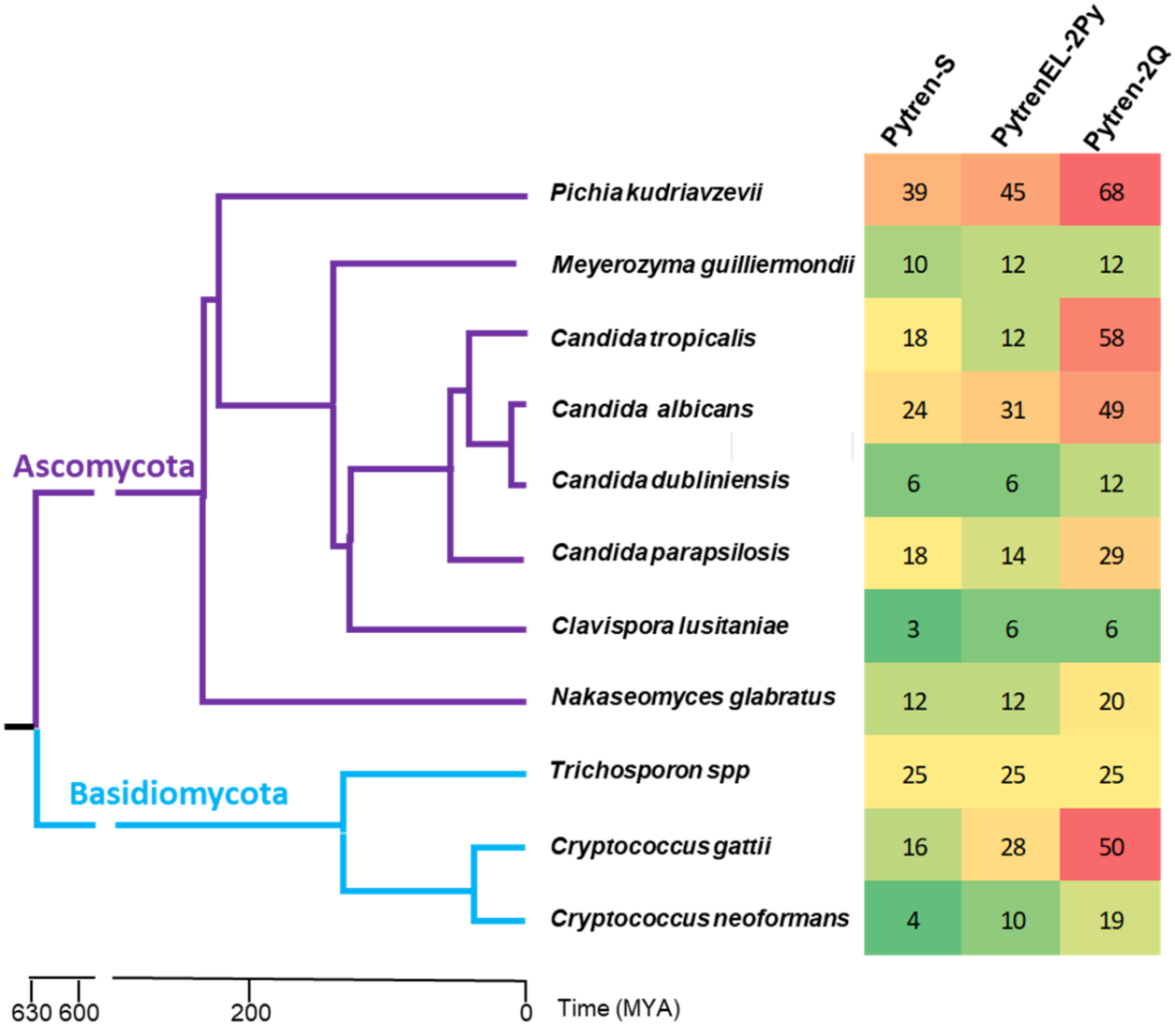
Heat map representing color-coded percent inhibition relative to drug control of the three most active compounds, Pytren-S, PytrenEL-2Py, and Pytren-2Q. Yeast pathogens are sorted by their phylogenetic similarities. MYA = million years ago.

### Potent Activity of Pytren-2Q against Pathogenic Molds

Sensitivity of molds to Pytren, Pytren-S, PytrenEl-2Py, Pytren-2QI, and Pytren-2Q was further tested in 23 clinical isolates, including one azole-resistant *A. fumigatus*, by using a microdilution method specific for molds. These isolates belong to 12 mold fungal pathogens, 6 of them included in the WHO priority groups. In addition, ATCC strains of *A. fumigatus*, *Aspergillus flavus*, and *Paecilomyces variotii* were included as controls (Figure 5 and ESI Table S2). Antifungal activity values were calculated as MIC_90_ (MIC causing inhibition of 90% of fungal growth) and MIC_100_ (MIC resulting in complete growth inhibition of the isolates). All compounds were able to reduce fungal growth by at least 90% compared to the untreated control, including species refractory to conventional antifungal drugs, such as *Fusarium solani*, *Lomentospora prolificans*, and *Scedosporium apiospermum*. Like in the disc diffusion and yeast microdilution assays, the most active compounds were Pytren-2Q, PytrenEl-2Py, and Pytren-S, while Pytren and Pytren-2QI had the lowest antifungal activity. Interestingly, Pytren-2Q was as effective as voriconazole against *A. fumigatus* WT isolates and 32 times more effective against its azole-resistant strain.

**Figure 5:**
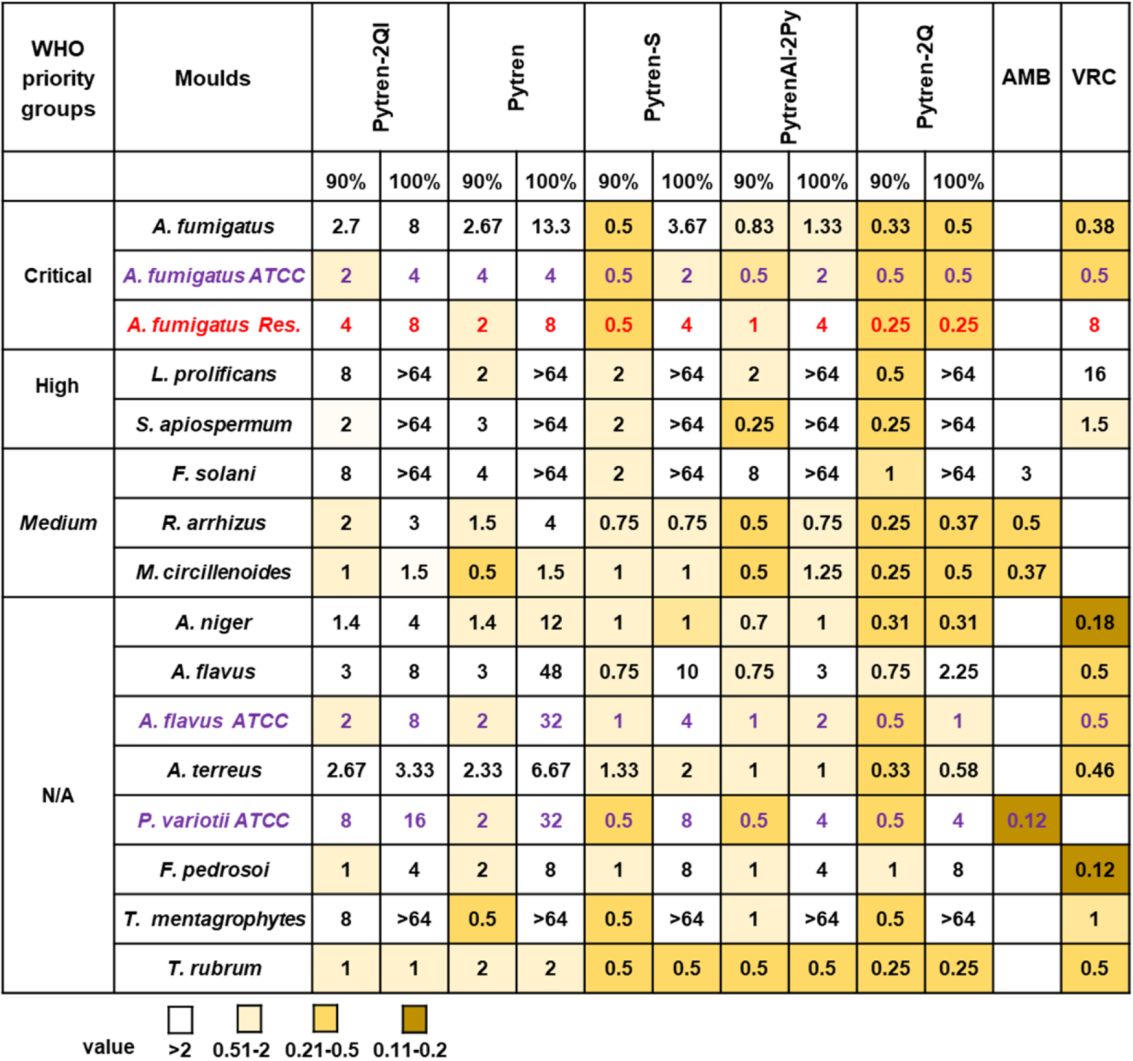
Antifungal activities of selected Pytren macrocyclic polyamines against human pathogenic molds. MICs in μg/mL of selected compounds Pytren, Pytren-S, PytrenEL-2Py, Pytren-2QI, and Pytren-2Q are depicted inside boxes. Values represent the MIC means from multiple isolates for each fungal species; the exact number of isolates tested is detailed in the Materials and Methods section. Mold strains are sorted in WHO critical, high, and moderate priority pathogens groups. To better visualize the results, a heat map was generated with colors ranging from MIC values >2 (white) to 0.11–0.2 (dark brown). Voriconazole (VRC) or amphotericin B (AMB) were used as controls.

When we plotted the percentage of activity relative to controls and sorted the mold pathogens by their phylogenetic relationships, we observed that, as in yeasts, sensitivity and resistance to Pytren-S, PytrenEl-2Py, and Pytren-2Q were strain specific and that phylogenetically close fungal pathogens showed different sensitivities, such as *Trichophyton mentagrophytes* and *Trichophyton rubrum*, or within the family *Aspergillaceae:* *Aspergillus terreus* and *A. flavus* (Figure 6). The most sensitive fungal pathogen was *T. rubrum* followed by the two mucorales strains, *Mucor circillenoides* and *R. arrhizus*; *T. mentagrophytes*; and the Hypocreomycetidae subclass representatives: *F. solani*, *L. prolificans*, and *S. apiospermum*. While Pytren-S, PytrenEl-2Py, and Pytren-2Q were sometimes even more effective than the control to stop growth at 90%, they did not completely stop fungal proliferation at 100%.

**Figure 6:**
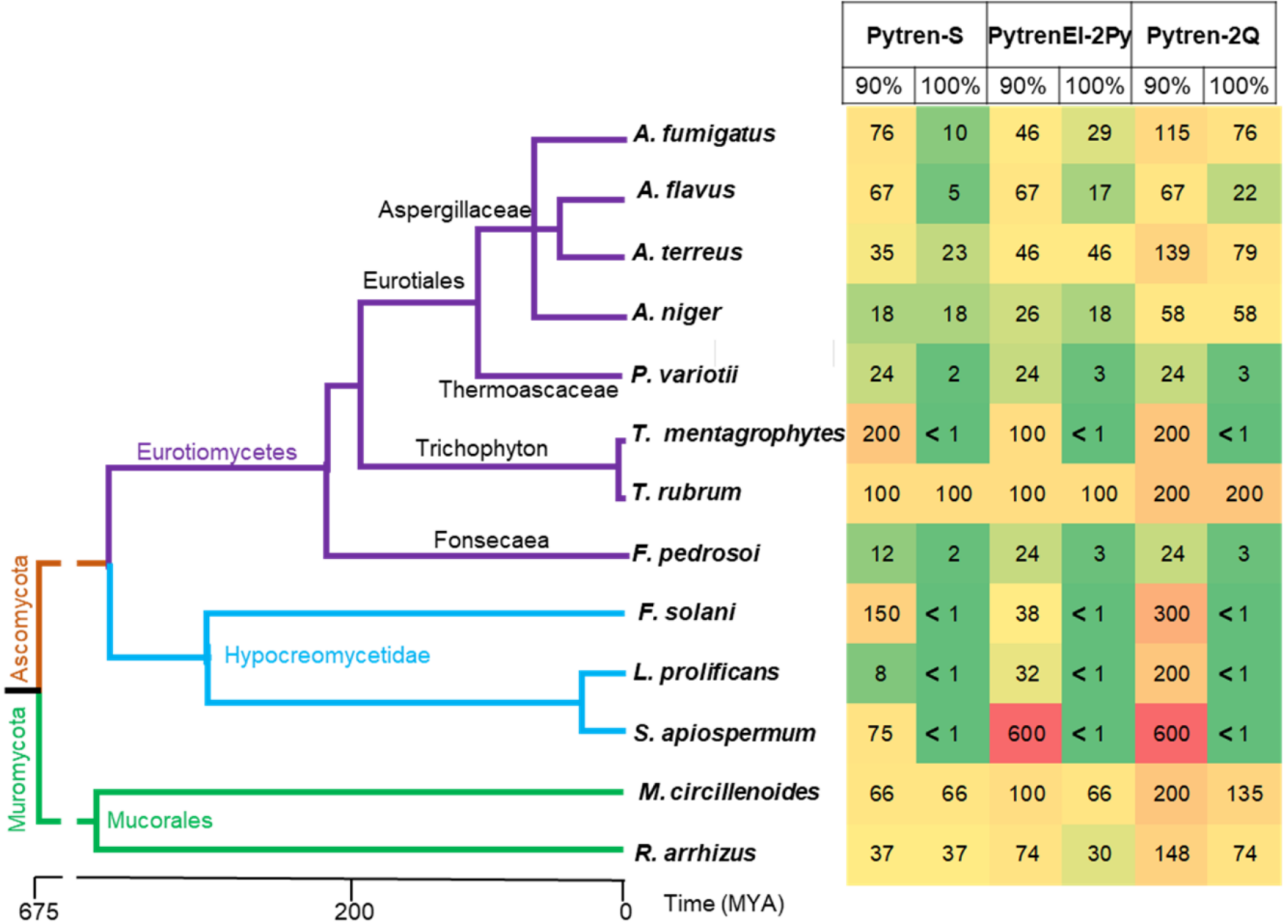
Heat map representing color-coded percent inhibition at 90% and 100% relative to the untreated control 100% of Pytren-S, PytrenEL-2Py, and Pytren-2Q. Mold pathogens are sorted by their phylogenetic similarities. MYA = million years ago.

We next assessed, using the checkerboard method, if drug interactions (synergy, additive effect, indifference, or antagonism) could occur between the two more active compounds against molds Pytren-2Q and PytrenEL-2Py and three common drugs with different mechanisms of action: voriconazole, amphotericin B, and caspofungin. We observed that Pytren-2Q and PytrenEl-2Py showed an additive effect with voriconazole or amphotericin B in *A. fumigatus*, and only Pytren-2Q with voriconazole in *M. circillenoides* (Table 1).

**Table 1:** Pytren-2Q and PytrenEL-2Py Additive Effect with Voriconazole or Amphotericin B against A. Fumigatus^a^,^b^,^c^,^d^

	Pytren-2Q			PytrenEl-2Py		
molds	VRC	CPF	AMB	VRC	CPF	AMB
*A. fumigatus*	0.53	1.5	0.51	0.53	1.5	0.51
*S. apiospermum*	2.01	1.5	2	1.00	2.5	3
*L. prolificans*	2	2	2	1	1.02	1
*M. circillenoides*	0.51	5	1	2.03	1	1.5
*F. solani*	2	2	1.25	2	2	2.03
*A. flavus*	1	1.5	1.25	1.17	1.12	1.01

a Synergy was defined as FICI ≤ 0.5.

b Additive effect when 0.5 < FICI < 1.0.

c Indifference when 1.0 ≤ FICI < 4.0.

d Antagonism when FICI ≥ 4.0.

To further investigate the mechanism of action, Pytren-2Q was tested in the presence of iron (1 mM FeSO_4_·7H_2_O). Under these conditions, the compound lost its antifungal activity, with MIC values ≥4 μg/mL for all tested isolates, suggesting that its efficacy is strongly dependent on iron chelation (data not shown).

Pytren-2Q was consistently the most active of the tested compounds with either screening method (diffusion disc or microdilution) and against both yeasts and molds. This noteworthy *in vitro* antifungal profile, particularly against molds including WT and azole-resistant strains of WHO critical priority *A. fumigatus*, in addition to the need for new antifungals, suggests that Pytren-2Q could be of pharmacological interest.

In this sense, Pytren-2Q is a small molecule (*M*_W_ = 374.16 g/mol), is highly water soluble (>0.4 g/mL), has good *in silico* pharmacokinetic properties, and shows low toxicity *in vitro* (Vero cells) and *in vivo* (BALB/c mice).^25^ This *in vitro* and *in vivo* low toxicity has also been reported for the antioxidant Mn^2+^ complexes of Pytren-2Q and its isomer Pytren-4Q.^18,26^

Although the antifungal mechanism of action of Pytren-2Q has not yet been thoroughly investigated, data generated in this study and in previous reports indicates that it could be related to PyTren-2Q metal-chelating properties.^17,19,27,28^ This hypothesis is supported by the following reasons: (i) Metal chelators are being used as antifungal drugs: to properly function, all living organisms, including pathogenic fungi, must regulate their intracellular levels of essential transition metals, such as iron, zinc, manganese, and copper. Effective assimilation and detoxification of these metals are necessary for pathogenic fungi to survive and proliferate within the infected host. In this sense, the mammalian host has developed the so-called “nutritional immunity”, which deals with both the essentiality and toxicity of these metals to defend itself against fungal and bacterial invasion by preventing fungal pathogens from acquiring these crucial micronutrients as an effective means of preventing their proliferation.^29–31^ This strategy has also been mimicked by topical or systemic iron chelator drugs such as deferasirox and deferiprone, which have shown antifungal efficacy against most human pathogenic fungi. Furthermore, iron chelators have also been successfully utilized in the clinical setting in combination therapies, augmenting the efficacy of existing antifungal drugs, and reducing the development of drug resistance.^31–33^ (ii) Most scorpiand-like macrocycles, including Pytren-2Q, are potent metal chelators. Pytren-2Q and its isomer, Pytren-4Q, among other scorpiand-like macrocycles, were tested for antiparasitic activity *in vitro* and in a murine model of the acute phase of Chagas disease, a parasitic infection caused by the protozoan *Trypanosoma cruzi*.^27^ Since the Fe-SOD enzymes of the parasites are druggable targets, the scorpiand-like macrocycles including Pytren-2Q and its isomer Pytren-4Q were assayed for their affinity toward iron in either its II or III oxidation state. The analysis showed that most scorpiand-like macrocycles form stable complexes with Fe^2+^ and Fe^3+^, with stability constants comparable to the ones reported for Cu^2+^.^27,34–36^ (iii) In the metal-chelating process, the side chain of the Pytren scorpiand macrocycle folds toward the macrocyclic core (herein the name “scorpiand”), thus forming a structural cage in where the metal gets trapped more or less tightly, depending on the metal characteristics and the nature of the heterocycle. The presence of the coordinating aliphatic amine between the macrocycle and heterocycle seems to be crucial to attaining this closed conformation. This could explain the lack of activity of PytrenMet-3py (L23) and Pyclen–OH, in which the aliphatic amine is fully substituted or nonexistent, further suggesting that the metal-chelating activity of the scorpiand macrocycle derivatives could be, at least in part, behind their antifungal activity. (iv) Moreover, although being isomers, Pytren-4Q is less active than Pytren-2Q, a fact that correlates with the inferior chelating activity of Pytren-4Q toward metals (related to the position of the quinoline nitrogen atom). Between Pytren-2Q and its isomer, Pytren-4Q, the former always forms the more stable complexes with metals and in the following order Cu^2+^ > Fe^3+^ > Zn^2+^ > Fe^2+^ > Mn^2+^ with log K values of 17.66, 16.93, 13.96, 10.06, and 8.91, respectively.^37^

Although our analyses have been carried out *in vitro* and have to be validated *in vivo*, our finding of a strong antifungal activity by Pytren-2Q, which was particularly active against pathogenic molds including WHO critical priority wild-type (WT) and azole-resistant *A. fumigatus*, is remarkable and, in our opinion, justifies future research efforts. Its favorable properties, such as low toxicity, water solubility, ease of synthesis, and additive effects, when combined with clinically used antifungal drugs, support its potential as a promising therapeutic candidate. In addition, we suggest that Pytren scorpiand macrocycles may provide a new type of structural scaffold that could be used as templates in the search for new antifungal agents. For these reasons, a patent (P202530332) has already been filed for the use of this family of compounds as antifungals.^38^

To guide future synthetic efforts and taking into consideration the data obtained for the critical pathogen *C. albicans*, structure–activity relationship (SAR) image (Figure 7) has been generated, summarizing the different structural aspects of the Pytren derivatives considered in this work.

**Figure 7:**
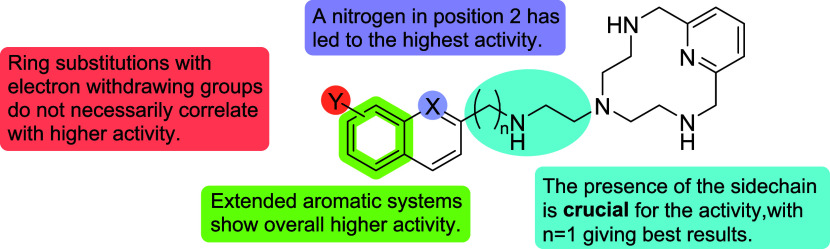
SAR figure for the Pytren scaffold considering the data of the activity against *C. albicans*.

## Materials and Methods

### Reagents

Phorbol 12-myristate 13-acetate (PMA), LPS (from *Escherichia coli* 0111: B4), l-ascorbic acid (L-ASC), and resveratrol (RSV) were supplied by Sigma-Aldrich (St. Louis, MO).

### Chemistry

A general synthetic route might be established for all of the ligands studied here, which consists of a reductive amination reaction between an amine functionality and the corresponding aromatic carboxaldehyde in ethanol to form the imine, or Schiff base, followed by the *in situ* reduction with sodium borohydride (Figure 8). The products are then precipitated and recrystallized with HCl, dried, stored, and used as hydrochloride salts.

**Figure 8:**
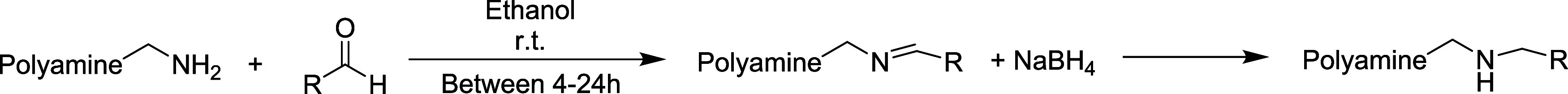
A scheme of the general synthetic route followed to functionalize the studied polyamine ligands.

Synthesis of compounds L1–L6 has already been described by Martín-Montes et al.^20^ Compounds L7 and L19 were first reported by Verdejo et al. in 2007 and L8 by Verdejo et al.^34,39^ Compound L9 has been described by Blasco et al. in 2010.^40^ L10 and L11 were first reported by Inclán et al. in 2014.^41^ L12 and L13 were reported by Clares et al. in 2011.^28^ L14 was first reported by Olmo et al. in 2014.^27^ Synthesis of compounds L15 and L16 has been published in a recently submitted article; we provide the details on their synthesis and characterization in the Supporting Information. L17 and L20 appear reported by Inclán et al. in 2012 ^35^ and L18 by Liberato et al. in 2017.^42^ L21 was first reported by Verdejo et al. in 2023.^43^ L22 and L23 first appear reported by Guijarro et al. in 2017.^44^ L24 was first published by Nebot-Guinot et al. in 2018.^45^ Finally, the control compound Pyclen–OH was reported by Lincoln et al. in 2013.^46^

### Strains, Isolates, and Cultures

A total of 57 clinical isolates (29 yeasts and 28 molds) were tested: *C. albicans* (5), *P. kudriavzevii* (formerly *C. krusei*) (4), *C. tropicalis* (4), *C. parapsilosis* (3), *N. glabratus* (formerly *C. glabrata*) (3), *M. guilliermondii* (formerly *C. guilliermondii*) (3), *C. dubliniensis* (1), *C. lusitaniae* (1), *Cryptococcus gattii* (2), Trichosporon spp. (1), *Aspergillus fumigatus* s.s (*A. fumigatus* sensu stricto) (5), *Aspergillus niger* (4), *Aspergillus flavus* (3), *Aspergillus terreus* (3), *R. arrhizus* (formerly *R. oryzae*) (2), *M. circinelloides* (2), *P. variotii* (1), *Fonsecaea pedrosoi* (1), *F. solani* (2), *T. mentagrophytes* (1), *S. apiospermum* (2), and *L. prolificans* (formerly *Scedosporium prolificans*) (1). Isolates were provided by the University of Chile (Chile) and the Faculty of Medicine of Reus (Spain), and the test panel included one *A. fumigatus* strain and one *N. glabratus* strain resistant to azoles. We used as control strains *C. parapsilosis* (Ashford) Langeron et Talice (ATCC 22019), *A. fumigatus* Fresenius (ATCC MYA-3626), *A. flavus* Link (ATCC 204304), and *P. variotii* (Udagawa et Suzuki) Houbraken et Samson (ATCC 22319).

### Antifungal Susceptibility Testing

The disc diffusion testing method was carried out using nonsupplemented Mueller–Hinton agar and 6 mm diameter paper discs containing 25 μg of the corresponding compounds dissolved in water. Control discs were loaded with 25 μg of fluconazole, 1 μg of voriconazole, or 20 μg of amphotericin B (CLSI m51-P). Broth microdilution methods specific for yeasts or molds were performed according to the guidelines of the Clinical and Laboratory Standards Institute (CLSI M-27 for yeasts and M38 for molds). Stock solutions of all tested compounds were prepared in water and subsequently diluted in an RPMI-1640 medium to obtain an initial concentration of 1280 μg/mL for use in the microdilution assays. Microdilution plates were prepared using 2-fold dilutions, yielding final drug concentrations ranging from 64 to 0.12 μg/mL for all of the compounds tested. MIC end points were determined visually by assessing turbidity relative to the drug-free growth control. Amphotericin B and voriconazole were used as the reference drugs. Drug interactions were assessed using the checkerboard method, and the fractional inhibitory concentration index (FICI) was used to classify drug interactions.^47^ Additionally, to elucidate whether the antifungal mechanism of action was mainly due to iron chelation, a solution of Pytren-2Q was enriched with a 1 mM solution of iron (Fe­(SO_4_)·7H_2_O) and tested following the same CLSI protocols.

## Supplementary Material



## Notes

Antifungal activities of Pytren, Pytren-S, PytrenEL-2Py, Pytren-2QI, and Pytren-2Q against human pathogenic yeasts and molds (extended tables). Synthesis and characterization data (NMR, mass spectrometry, and elemental microanalysis) of L15 and L16 (PDF)

## Abbreviations

WHO: World Health Organization
IFD: invasive fungal disease
WT: wild type
MIC: minimum inhibitory concentrations
