# Supplementary material for: Significant Activity of Pytren-2Q, a 2‑Quinoline Polyamine Compound, against High-Concern Human Pathogenic Fungi
Source: ACS Omega. 2026 Feb 6;11(6):9547–56. doi: 10.1021/acsomega.5c09700 (PMC12917647; doi:10.1021/acsomega.5c09700)
Supplement: Supplementary file 1 [file ao5c09700_si_001.pdf]

Electronic Supplementary Information

Significant activity of Pytren-2Q, a 2-quinoline polyamine compound, against high-concern human pathogenic fungi.

Mario Inclán,[a,b,] Maria Paz Clares,[a] Eduardo Álvarez-Duarte,[c] Fabiola Fernández-Silva, [d] Valentina Salas,[e] Josep Guarro,[f] Javier U. Chicote,[g] Begoña Verdejo,[a] Estefanía Delgado-Pinar,[a] Enrique García-España,[a,*] Antonio García-España,[h,*] Enrique Calvo.[i*]*

[a] Molecular Science Institute, Universitat de València, C/ Catedrático José Beltrán 2, 46980, Paterna, Spain.

[b] Departamento de Farmacia, Facultad de Ciencias de la Salud, Universidad CEU Cardenal Herrera, Carrer Santiago Ramón y Cajal, 20, 46113, Alfara del Patriarca, Spain.

[c] Laboratorio Micología, ICBM – F. de Medicina, Universidad de Chile, 1025000, Santiago Metropolitan Region, Chile.

[d] Instituto Microbiología Clínica, Facultad de Medicina, Universidad Austral de Chile, Independencia 631, Valdivia, Chile.

[e] Mycology Laboratory, Biomedical Department, Public Health Institute of Chile, Av. Marathon 1000, Santiago, Chile.

[f] Unitat de Microbiologia, Departament de Ciències Mèdiques Bàsiques, Facultat de Medicina i Ciències de la Salut, Universitat Rovira i Virgili and Institut d'Investigació Sanitària Pere Virgili (IISPV). Carrer Sant Llorenç, 21, 43201, Reus, Tarragona, Spain.

[g] Departament de Medicina i Cirurgia, Institut d'Investigació Sanitària Pere Virgili (IISPV), Servei Anatomia Patològica, Hospital Universitari Joan XXIII Universitat Rovira i Virgili (URV), Mallafré Guasch, 4, 43007 Tarragona, Spain.

[h] Departamento de Bioquímica y Biología Molecular, Universitat de Valencia, Carrer del Dr. Moliner, 50, 46100 Burjassot, Spain.

[i] Department of Biochemistry and Biotechnology; Institute of Health Research Pere Virgili (IISPV); Center of Environmental, Food and Toxicological Technology (TecnATox), Universitat Rovira i Virgili, C/ Marcel·lí Domingo 1, 43007, Tarragona, Spain.

Table S1. Antifungal activities of selected Pytren macrocyclic polyamines against human pathogenic yeasts. Yeast strains are sorted in critical, high, and moderate priority groups as in the WHO list of fungal pathogens. The table includes the range and the number of isolates.

WHO priority groups	Yeasts (n° isolates)		Pytren	Pytren-S	PytrenAl-2Py	Pytren-2Q	Pytren-2QI	VRC
Critical priority group	*Candida albicans (5)*	Range	2-8	0.5-1	0.5-1	0.25-1	0.5-2	0.12-0.5
		GM	4	0.9	0.7	0.45	1.1	0.22
	Criptococcus neoformans (2)	Range	1-2	1-2	0.25-1	0.12-0.5	1-2	0.06
		GM	1.5	1.5	0.62	0.31	1.5	0.06
High priority group	*Candida tropicalis (4)*	Range	1-2	1-2	1	0.25-0.5	2-4	0.12-0.25
		GM	2	1	1	0.31	2.5	0.18
	Candida parapsilosis (2)	Range	1-2	1-2	0.5-1	0.25-0.5	1-4	0.12
		GM	1.67	1.67	0.83	0.42	2.33	0.12
	C. parapsilosis ATCC 22019 (1)	MIC	1	1	0.5	0.5	1	0.12
	Nakaseomyces glabratus (2)	Range	1	1	1	0.5-1	1-2	0.12
		GM	1	1	1	0.67	1.33	0.12
	N. glabratus Resistant isolate (1)	MIC	1	1	1	1	2	8
Medium priority	*Pichia kudriavzevii (4)*	Range	2-4	0.5-1	0.5-1	0.25-1	1-2	0.12-0.5
		GM	2.5	0.87	0.75	0.5	1.5	0.34

group	*Criptococcus gattii (2)*	Range	2-4	2	0.25-2	0.25-1	1-4	0.12-0.5
		GM	3	2	1.12	0.62	2.5	0.31
N/A	*Meyerozyma g uilliermondii (3)*	Range	1-4	1	0.5-1	0.5-1	2-4	0.06-0.12
		GM	2.33	1	0.83	0.83	2.67	0.1
	Clavispora lusitanea (1)	MIC	1	1	0.5	0.5	2	≤0.03
	Candida dubliniensis (1)	MIC	2	1	1	0.5	2	0.06
	Trichosporon spp (1)	MIC	2	1	1	1	2	0.25

Table S2. Antifungal activities of selected Pytren macrocyclic polyamines against human pathogenic molds. MICs in µg/mL of selected compounds Pytren, Pytren-S, PytrenEL-2Py, Pytren-2QI, and Pytren-2Q are depicted inside boxes. Values represent the MIC means from multiple isolates for each fungal species; the exact number of isolates tested is shown in parenthesis. Mold strains are sorted in WHO critical, high, and moderate priority pathogens groups. Voriconazole (VRC) or amphotericin B (AMB) were used as controls.

WHO priority groups	Moulds (nº isolates)		Pytren		Pytren-S		PytrenAl-2Py		Pytren-2Q		Pytren-2QI		AMB	VRC
		%	90	100	90	100	90	100	90	100	90	100		
Critical priority group	*Aspergillus fumigatus* (3)	Range	2-4	4-32	0.5	1-8	0.5-1	1-2	0.25-0.5	0.25-1	2-4	4-16		0.12-0.5
		GM	2.67	13.3	0.5	3.67	0.83	1.33	0.33	0.5	2.7	8		0.38
	A. fumigatus ATCC 3626 (1)	MIC	4	4	0.5	2	0.5	2	0.5	0.5	2	4		0.5
	A. fumigatus Resistant (1)	MIC	2	8	0.5	4	1	4	0.25	0.25	4	8		8
High priority group	*Scedosporium prolificans* (1)	MIC	2	>64	2	>64	2	>64	0.5	>64	8	>64		16
	Scedosporium apiospermum (2)	Range	2-4	>64	2	>64	0.25	>64	0.25	>64	2	>64		1-2
		GM	3	>64	2	>64	0.25	>64	0.25	>64	2	>64		1.5
Medium priority group	*Fusarium solani* (2)	Range	4	>64	2	>64	8	>64	1	>64	8	>64	2-4	
		GM	4	>64	2	>64	8	>64	1	>64	8	>64	3	
	Rizhopus arrhizus (2)	Range	1-2	4	0.5-1	0.5-1	0.5	0.5-1	0.25	0.25-0.5	2	2-4	0.5	
		GM	1.5	4	0.75	0.75	0.5	0.75	0.25	0.37	2	3	0.5	
	Mucor circillenoides (2)	Range	0.5	1-2	1	1	0.5	0.5-2	0.25	0.5	1	1-2	0.25-0.5	
		GM	0.5	1.5	1	1	0.5	1.25	0.25	0.5	1	1.5	0.37	
N/A	*Aspergillus niger* (4)	Range	1-2	8-16	1	1	0.5-1	1	0.25-0.5	0.25-0.5	1-2	4		0.12-0.25
		GM	1.4	12	1	1	0.7	1	0.31	0.31	1.4	4		0.18
	Aspergillus flavus (2)	Range	2-4	32-64	0.5-1	4-16	0.5-1	2-4	0.5-1	0.5-4	2-4	8		0.5
		GM	3	48	0.75	10	0.75	3	0.75	2.25	3	8		0.5
	A. flavus ATCC 204304 (1)	MIC	2	32	1	4	1	2	0.5	1	2	8		0.5
	Aspergillus terreus (3)	Range	1-4	2-16	1-2	1-4	0.5-2	0.5-2	0.25-0.5	0.25-1	2-4	2-4		0.12-1
		GM	2.33	6.67	1.33	2	1	1	0.33	0.58	2.67	3.33		0.46
	Paecilomyces variotii ATCC 36257 (1)	MIC	2	32	0.5	8	0.5	4	0.5	4	8	16	0.12	
	Fonsecaea pedrosoi (1)	MIC	2	8	1	8	1	4	1	8	1	4		0.12
	Trichophyton mentagrophytes (1)	MIC	0.5	>64	0.5	>64	1	>64	0.5	>64	8	>64		1
	Trichophyton rubrum (1)	MIC	2	2	0.5	0.5	0.5	0.5	0.25	0.25	1	1		0.5

Synthesis of Pytren-2QI (L15). 6-(2-Aminoethyl)- 3,6,9-triaza-1(2,6)-pyridinacyclodecaphane (0.58 g, 2.32 mmol) and 8-iodoquinoline-2-carbaldehyde (0.45 g, 2.35 mmol) are dissolved in 100mL of dry ethanol and stirred at room temperature for 2 h. Then, sodium borohydride (0.9 g, 23 mmol) is added, and the bulk is stirred for 2 h more. The solution is vacuum evaporated and extracted with $CHCl_3/H_2O$ (6x100). The organic phase is taken to dryness and dissolved in dry ethanol. The hydrochloride salt of the product is precipitated by adding a concentrated hydrochloride acid solution. Yield: 74% ^{1}H NMR (300 MHz, D_2O) δ 8.51 (d, *J* = 7.6 Hz, 1H), 8.40 (d, *J* = 8.5 Hz, 1H), 8.04 (t, 1H), 7.99 (d, *J* = 8.0 Hz, 1H), 7.57 (d, *J* = 8.5 Hz, 1H), 7.48 (d, *J* = 8.0 Hz, 2H), 7.45 (t, *J* = 7.6 Hz, 1H), 4.77 (s, 2H), 4.66 (s, 4H), 3.65-3.60 (m, 2H), 3.37-3.29 (m, 6H), 3.04-3.00 (m, 4H). ^{13}C NMR (126 MHz, D_2O) δ 152.3,148.9, 140.9, 139.8, 139.2, 129.2, 128.5, 128.3, 122.2, 120.4, 101.5, 51.3, 50.6, 49.4, 45.9, 42.9. Elemental analysis: calculated for $C_{23}H_{26}N_6I{\cdot}4HCl{\cdot}4H_2O{\cdot}0.4(C_4H_8O_2)$: C: 38.54%; N:10.96%; H: 5.42%. Found: C: 38.54%; N:11.02%; H: 5.08%. No melting temperature has been registered since the compound decomposes without melting.

Figure S1. ^{1}H and ^{13}C NMR spectra of compound L15 with suggested signal assignation.

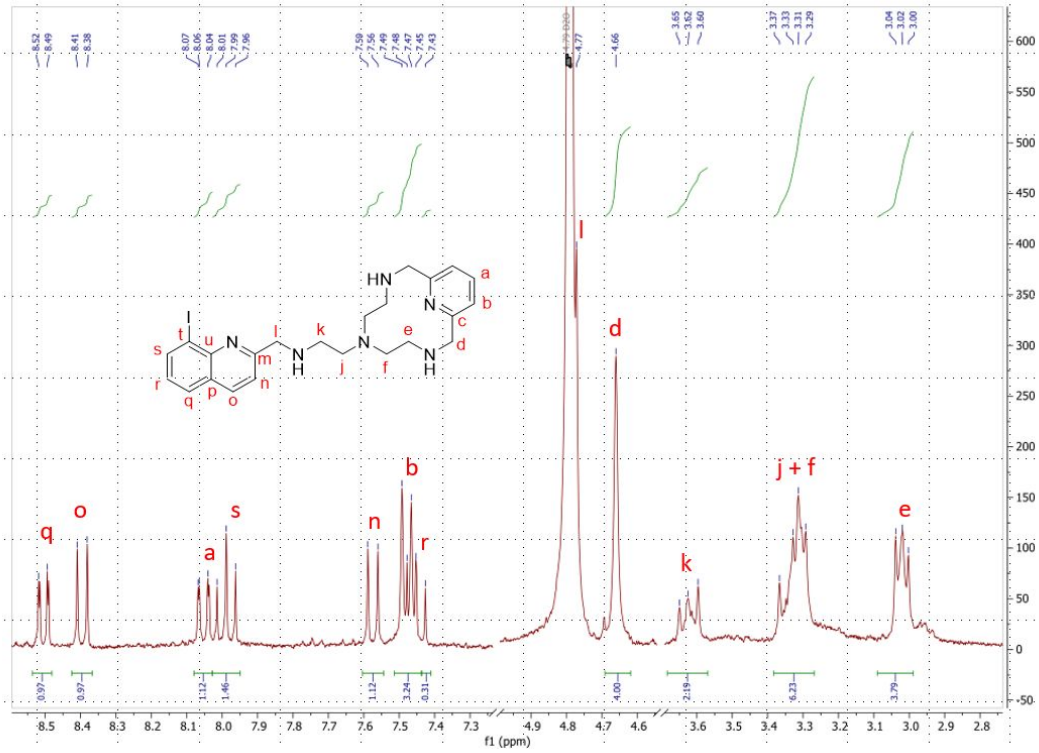

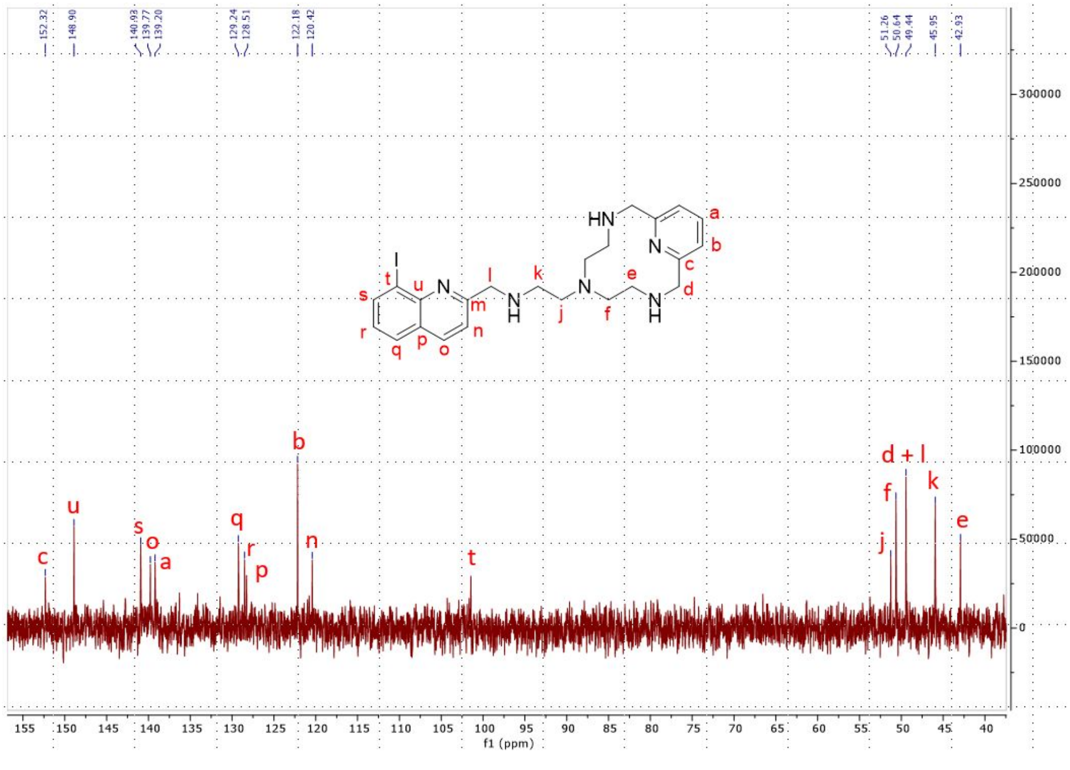

Synthesis of Pytren-2QCl (L16). The same procedure applies for the synthesis of Pytren-2QCl, but 8-chloroquinoline-2-carbaldehyde[1] is used instead of 8-iodoquinoline-2-carbaldehyde Yield: 68% ^{1}H NMR (300 MHz, D_2O) δ 8.38 (d, *J* = 8.5 Hz, 1H), 7.89 – 7.83 (m, 3H), 7.55 – 7.48 (m, 2H), 7.35 (d, *J* = 8.0 Hz, 2H), 4.64 (s, 2H), 4.54 (s, 4H), 3.48 – 3.43 (m, 2H), 3.21-3.13 (m, 6H), 2.88-2.85 (m, 4H). ^{13}C NMR (75 MHz, D_2O) δ 151.9, 148.8, 142.5, 139.7, 139.4, 131.4, 130.8, 129.1, 127.6, 122.1, 121.0, 51.0, 50.5, 49.4, 46.0, 43.2. Elemental analysis: calculated for $C_{23}H_{26}N_6Cl{\cdot}4HCl{\cdot}2H_2O$: C: 45,75%; N:13.92%; H: 5.67%. Found: C: 45.84%; N:14.02%; H: 5.26%. No melting temperature has been registered since the compound decomposes without melting.

Figure S2. ^{1}H and ^{13}C NMR spectra of compound L16 with suggested signal assignation.

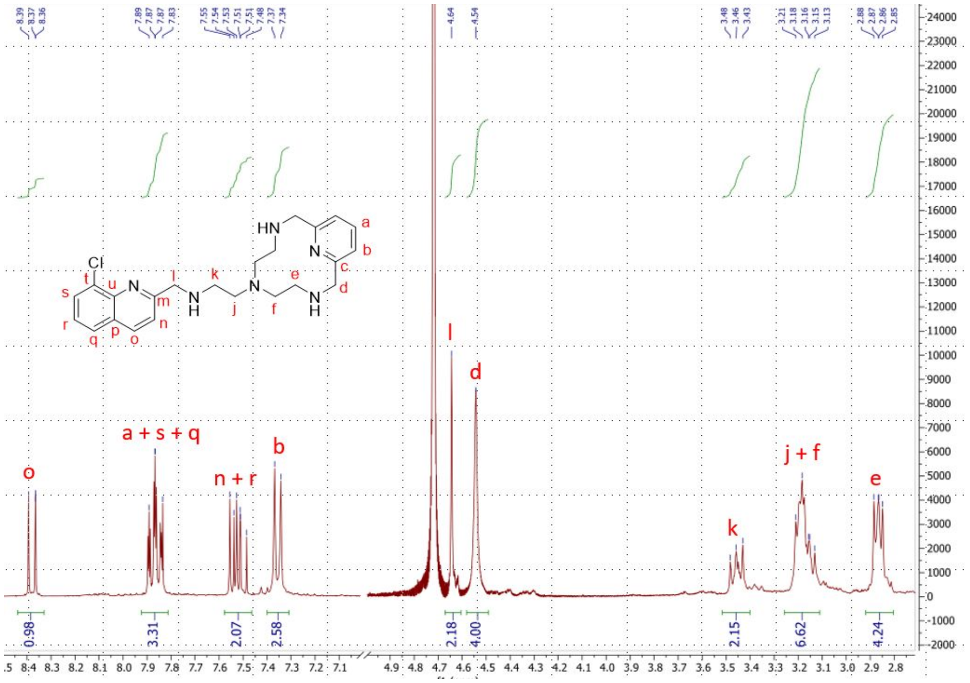

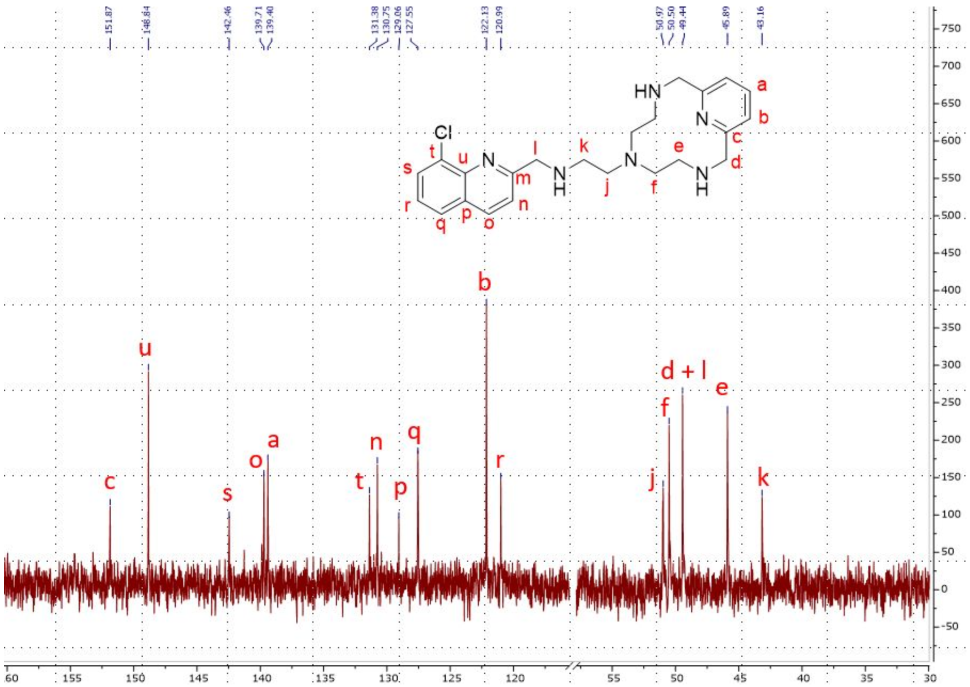

Figure S3. ^{1}H and ^{13}C NMR spectra of compound Pytren-2Q (L12).

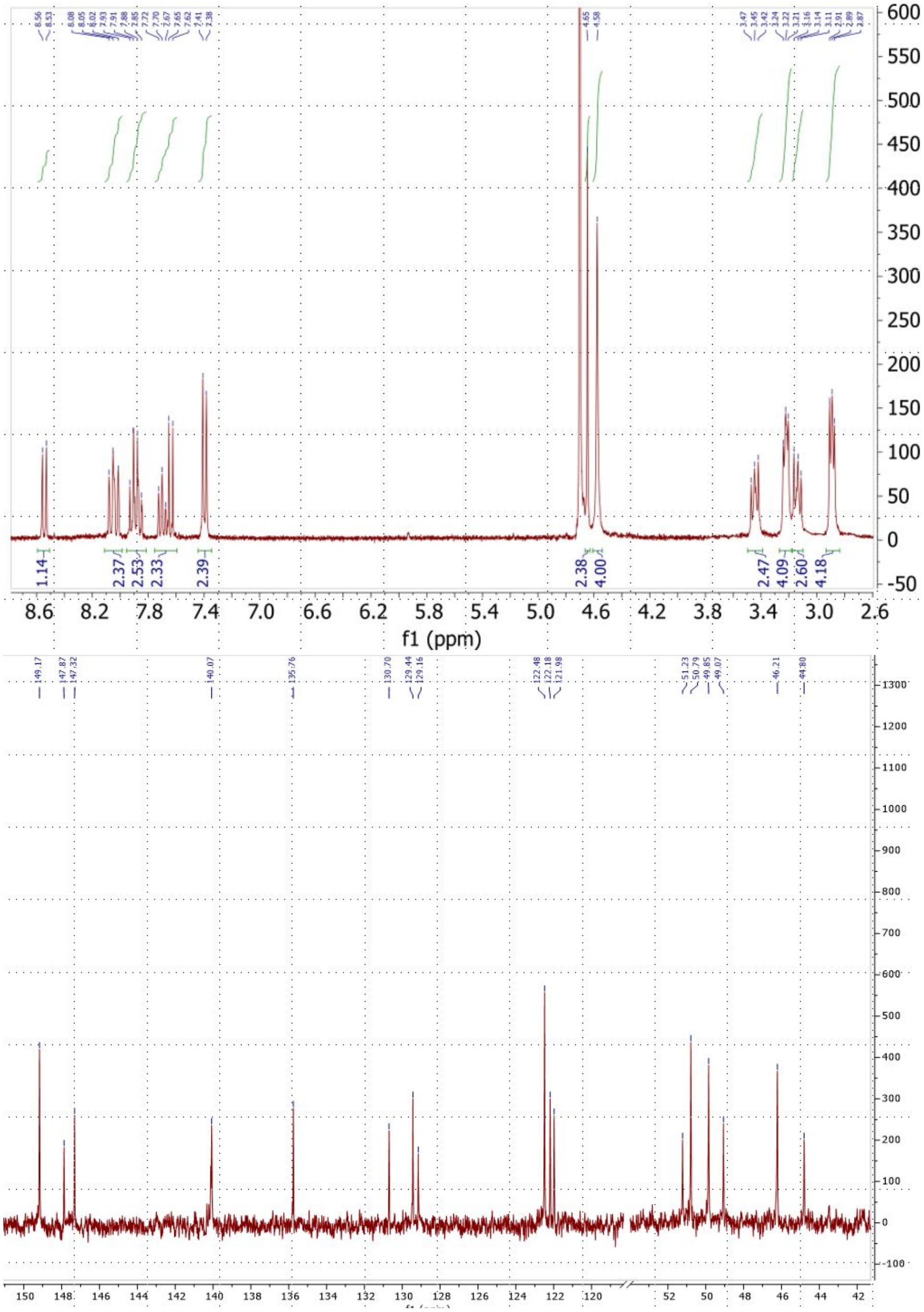

(1) Ballesteros-Garrido, R.; Leroux, F.; Ballesteros, R.; Abarca, B.; Colobert, F. The Deprotonative Metalation of [1,2,3]Triazolo[1,5-a]Quinoline. Synthesis of 8-Haloquinolin-2-Carboxaldehydes. *Tetrahedron* **2009**, *65*, 4410–4417.
